# Supplementary material for: Intranasal delivery of Thyroid hormones in MCT8 deficiency
Source: PLoS One. 2020 Jul 20;15(7):e0236113. doi: 10.1371/journal.pone.0236113 (PMC7371167; doi:10.1371/journal.pone.0236113)
Supplement: Attachment — Submitted filename: Response to Reviewers.docx [file pone.0236113.s001.docx]

Dear Dr Iglesias Osma,

We have revised the manuscript reflecting all the Reviewers concerns. We believe we have met all Reviewers’ and journal requests and therefore we look forward to the manuscript being now considered for publication in Plos One.

Sincerely Yours,

Ana Guadaño-Ferraz

Corresponding Author

[aguadano@iib.uam.es](mailto:aguadano@iib.uam.es)

1. Please ensure that your manuscript meets PLOS ONE's style requirements, including those for file naming. The PLOS ONE style templates can be found at https://journals.plos.org/plosone/s/file?id=wjVg/PLOSOne_formatting_sample_main_body.pdf and https://journals.plos.org/plosone/s/file?id=ba62/PLOSOne_formatting_sample_title_authors_affiliations.pdf

Already done.

2. At this time, we request that you please report additional details in your Methods section regarding animal care, as per our editorial guidelines:

(1) Please state whether the provided ethics committee contains animal welfare experts or whether an animal ethics or IACUC committee reviewed and approved the study. Please provide the full name of the committee that reviewed and approved the study.

More details regarding the review boards are now included in lines 111-114 (revised manuscript).

(2) Please include the method of euthanasia.

We already mentioned that mice were anesthetized and then transcardially perfused, but we have now included the sentence “All animals were euthanized by transcardial perfusion under anesthesia 15 minutes after the last administration…” to make the method of euthanasia more clear.

(3) Please provide the source of the animals used in the study.

Animals were provided by Dumitrescu and colleagues [22] and by Schneider and colleagues [48] in C57BL/6J and a colony was created at our animal facility, we do not use external suppliers. This is already stated in lines 125-127 (revised manuscript).

Please use the space provided to explain your answers to the questions above. You may also include additional comments for the author, including concerns about dual publication, research ethics, or publication ethics. (Please upload your review as an attachment if it exceeds 20,000 characters)

**Reviewer #1:** This manuscript reports a study of the effectiveness of intranasal delivery of thyroid hormone in the treatment of monocarboxylate transporter 8 (MCT8) deficiency. Loss of function mutations in MCT8 in humans lead to severe neurodevelopmental defects, apparently due to impairment in the transport of thyroid hormone across the blood brain barrier. Although these mutations result in hypothyroidism in the brain they produce hyperthyroidism in peripheral tissues. This manuscript describes a test of the ability of intranasal delivery of thyroid hormone as a means to bypass the brain blood barrier and selectively correct the hypothyroid neurological phenotype.

The authors tested intranasal delivery of T4 or T3 thyroid hormone in WT, MCT8 deficient, and double MCT8-/DIO2 deficient mice. Although the intranasal route greatly increased plasma T3 or T4 levels compared to controls, no, or only statistically non-significant increases in hormone in cerebral cortex in the MCT8 deficient animals were detected. Given the further increases in plasma thyroid hormone levels observed with intranasal administration were non-desirous, the authors also tested hormone formulations containing BSA (which binds T3 and T4) with or without phenylephrine (PHE) a vasoconstrictor. Neither formulation diminished the rise in serum thyroid hormone observed after intranasal administration. The authors conclude that their “data suggests intranasal delivery of thyroid hormones is not a suitable therapeutic strategy for MCT8 deficiency, although alternative formulations could be considered in the future to improve the nose-to-brain transport.”

The experiments described here appear to be well designed and carefully performed. Appropriate controls were employed, a variety of approaches were tested, the results are definitive, and the authors’ conclusions are convincing. Given that MCT8 deficiency in humans creates severe neurodevelopmental defects, the clinical need for exploring methods to ameliorate this defect is clear. Further, the authors’ approach to this goal was logical: intranasal delivery has been demonstrated previously as a non-invasive route that can, for several drugs, bypass the blood brain barrier. Although the ultimate results reported here were negative, i.e. that the intranasal route was not successful, this study is nonetheless valuable both for demonstrating that the intranasal approach as formulated here is not effective (important information for other scientists who might otherwise be tempted to try the same approach) and as a foundation for future approaches that may prove more useful. Very few specific concerns were noted:

1. All studies here assayed cerebral cortex levels of T3 or T4 thyroid hormone 15 minutes after administration. Why did the authors choose 15 mins. and has a more extensive time course been examined?

Falcone et al., (2014) provided a very complete time course of brain concentration of albumin after intranasal administration at different time points (5 min, 10 min, 30 min, 60 min, 2 hrs, 4 hrs and 6 hrs). They found that in the frontal, parietal and occipital cortex, the concentration of albumin was highest at 5 minutes, remained steady in the frontal and occipital cortex through the first 2 hours, while in the parietal cortex decreased earlier, having undetectable levels by 2 hours. Based on this, as our measurements were performed in the whole cerebral cortex, 15 minutes following the last administered drop should have resulted in an increase of THs in the cerebral cortex. We now include this in lines 157-159 (revised manuscript).

The most relevant finding of this study is that TH levels in plasma increase substantially following intranasal administration, and therefore does not seem to be suitable for treatment of MCT8 deficiency. Had we found no or mild increases in TH plasma levels following intranasal administration, we would have certainly performed TH measurements in more brain regions, and we would have performed a more extensive time course. Unfortunately, the high increase in plasma TH levels after intranasal administration drove us to try to prevent TH from reaching the bloodstream rather than expanding the brain regions or the time course.

2. An interesting question is if counteracting the brain hypothyroidism in adults is likely to substantially ameliorate (at least in part) the neurological defects, or if the MCT8 defect may already have produced irreparable damage by the fetus/neonate stages. The authors should include a discussion of this issue.

This is a very interesting point and a key question to be addressed in the MCT8 deficiency field. All evidence suggests than an early treatment would be more beneficial to prevent brain damage during the neonate stages rather than to restore brain impairments later in life. This in now discussed in lines 370-373 (revised manuscript).

3. The authors may wish to consider presenting their data as box-whisker plots, which would better visualize the statistics involved, rather than as columns; however this is not a requirement.

We agree that box-whisker plots provide a lot of information, so we have followed the reviewer’s suggestion.

**Reviewer #2:** In the present manuscript, Grijota-Martínez et al. attempt to intranasally deliver thyroid hormones to the CNS to potentially treat centrally induced deficiencies associated with MCT8. My major concern is with the authors' conclusion that they are not able to increase thyroid hormones in the brain through the intranasal route. It seems that the experiments were not sufficiently powered to draw this conclusion. Data generated from the intranasal route is typically more variable than data generated by other routes of administration. The conclusion that intranasal administration of T4 does not increase brain levels based on an n=2 or 3 at a single 15 minute time point is not convincing. Two or three animals per treatment group is rarely sufficient to draw a statistically significant conclusion, especially with the intranasal route. The data shows a trend towards increased T4 in cortex after intranasal administration. This increase would likely be statistically significant if the study was not so underpowered. An n=6 animals per treatment group for the studies described in Figure 1 would be much more convincing. There is simply not enough data currently in the manuscript to conclude that intranasal delivery of thyroid hormones does not result in delivery to the CNS.

The conclusions obtained in this study are not obtained from the experiment (Figure 1) the reviewer is referring to. It is stated in the manuscript that this was a pilot study aimed to determine if THs significantly entered the blood following intranasal delivery. Indeed, even though intended as a pilot study, the data obtained from it was powered enough to support this finding (Plasma T4 levels). According to the power calculation tool of The Experimental Design Assistant (EDA) of the NC3Rs with a significance threshold (α) of 0.05 and statistical power (1-β) of 0.95, and introducing the largest variability (SD= ±8.121) and the lowest effect (and increase of 82ng/ml between Wt and Wt+T4) observed, the recommended number of animals is n=2.

As for the data regarding T4 content in the cerebral cortex, we agree with the reviewer that with a larger n, this would have resulted in a statistically significant increase in brain T4. However, and as already stated in the manuscript, this finding would not have provided any useful information as it would not have been possible to differentiate if this increase in brain T4 would have been mediated by direct nose-to-brain transport or by indirect transport through the OATP1C1 transporter at the blood brain barrier. Following animal ethics principles, further studies aimed to increase the n of animals were not performed, as this would not have provided useful data. Nevertheless, we understand the reviewer’s concern regarding this figure and the data related to it, so we have decided to delete it from the manuscript.

The conclusions from this study are drawn from the rest of the data, which is sufficiently powered.

**Reviewer #3:** In this manuscript the authors describe studies where T4 and T3 were administered intranasally to mouse models lacking the thyroid hormone transporter MCT8 to determine if this could be an effective strategy to increase thyroid hormone (TH) levels in the brain. They also assessed and if this led to changes in circulating TH, and evaluated other strategies such as binding T3 with BSA or use of the vasoconstrictor phenylephrine to prevent TH spillover into the circulation. This study has important clinical applications since patients with MCT8 mutations have impaired TH transport across the blood brain barrier which leads to severe and devastating neurological impairment. Although the authors do not find that any of the protocols described are an effective strategy to alter TH levels in either of the MCT8KO models used, they present a strong starting point towards future studies which could lead to important therapies. These studies are clearly presented and the introduction and discussion are well written, informative, and balanced with the limitations considered and acknowledged.

1. In the discussion the authors elaborate on the rationale behind the dosages used but can they also discuss why they chose the timing that was used? Have they looked at TH content (or also possibly expression of TH-regulated genes) in the brain at later time points after administration?
Reviewer 1 has raised the same point. Falcone et al., (2014) provided a very complete time course of brain concentration of albumin after intranasal administration at different time points (5 min, 10 min, 30 min, 60 min, 2 hrs, 4 hrs and 6 hrs). They found that in the frontal, parietal and occipital cortex, the concentration of albumin was highest at 5 minutes, remained steady in the frontal and occipital cortex through the first 2 hours, while in the parietal cortex decreased earlier, having undetectable levels by 2 hours. Based on this, as our measurements were performed in the whole cerebral cortex, 15 minutes following the last administered drop should have resulted in an increase of THs in the cerebral cortex. We now include this in lines 157-159 (revised manuscript).

Regarding the second question, the most relevant finding of this study is that TH levels in plasma increase substantially following intranasal administration, and therefore does not seem to be suitable for treatment of MCT8 deficiency. Had we found no or mild increases in TH plasma levels following intranasal administration, we would have certainly performed TH measurements in more brain regions, and we would have performed a more extensive time course to measure TH content and gene expression of TH target genes. However, the lack of increase of TH content in the cerebral cortex and the high increase in plasma TH levels after intranasal administration drove us to prioritize preventing TH from reaching the bloodstream rather than expanding the time course and its corresponding analysis (TH measurements and T3-target genes expression).

2. In Fig. 2. why is the student’s t-test between saline treated WT and Mct8/Dio2KO also added in? The two-way ANOVA plus Bonferroni’s post test is more appropriate.

With the t-test between saline treated Wt and *Mct8/Dio2*KO, we wanted to illustrate that saline-treated *Mct8/Dio2*KO mice display the increase in T3 plasma levels in basal conditions characteristic of MCT8 deficiency. The reviewer is right to point that it is not necessary, so we have now deleted this comparison.

3. While these strategies may not have prevented spillover into the circulation they still may have facilitated uptake into the brain. Do the authors have any data in this regard?

We did not perform TH measurements in the brain in these animals because, as they are Wt animals and they present the MCT8 transporter at the BBB, even if we had found an increase in T3 content in the brain, we would not have been able to differentiate if this was being mediated by direct intranasal uptake or indirectly from the bloodstream. In these experiments we were aiming to prevent T3 from reaching the blood. If this had been achieved, we would have then performed TH measurements in the brain and we would have also repeated the studies in *Mct8/Dio2*KO animals. Unfortunately, this was not the case.

4. Minor point: Figure 3 doesn’t have statistical differences indicated.

Thank you, these have now been included.

5. Lines 354-358. I don’t follow the logic of this section. If 0.01% to 1% of 5 micrograms of T3 was estimated to be delivered wouldn’t 0.5 to 50 ng to be delivered to both genotypes? Why are the estimated increases different between Wt and Mct8/Dio2KO? Please clarify.

Yes, 0.5 to 50 ng would be delivered in both genotypes. However, we wanted to illustrate the increase in T3 content this 0.5 to 50 ng would represent. The reviewer is right, we should express it in fold change.

This will be different between genotypes as, in basal conditions, Wt animals have an average of 4 ng/g of T3 in the cerebral cortex while *Mct8/Dio2*KO animals have an average of 1 ng/g. The approximate weight of a mouse brain of that strain, sex, age and weight is 0.5g, this represents a total content of 2 ng of T3 in Wt mice and a total content of 0.5 ng of T3 in *Mct8/Dio2*KO animals. Based on this, this would represent a 1.25- to 25-fold increase in T3 brain content in a Wt animal and a 2- to 100-fold increase in T3 brain content in a *Mct8/Dio2*KO mouse. This has now been changed (lines 401-405 revised manuscript).
